# Cell–Cell Interaction Proteins (Gap Junctions, Tight Junctions, and Desmosomes) and Water Transporter Aquaporin 4 in Meningothelial Cells of the Human Optic Nerve

**DOI:** 10.3389/fneur.2017.00308

**Published:** 2017-06-29

**Authors:** Thi Ngoc Co Zeleny, Corina Kohler, Albert Neutzner, Hanspeter E. Killer, Peter Meyer

**Affiliations:** ^1^Department of Ophthalmology, Kantonsspital Aarau, Aarau, Switzerland; ^2^Department of Biomedicine, Ocular Pharmacology and Physiology, University Hospital Basel, Basel, Switzerland; ^3^Department of Ophthalmology, University Hospital Basel, Basel, Switzerland

**Keywords:** optic nerve degeneration, optic nerve microenvironment, meninges, meningothelial cells, cell–cell interactions, aquaporin 4, cerebrospinal fluid homeostasis

## Abstract

**Purpose:**

Meningothelial cells (MECs) play a central role in the maintenance of cerebrospinal fluid (CSF) homeostasis and in physiological and pathophysiological processes within the subarachnoid space (SAS) linking them to optic nerve (ON) pathologies. Still, not much is known about their structural properties that might enable MECs to perform specific functions within the ON microenvironment.

**Methods:**

For closer characterization of the structural properties of the human MEC layer in the arachnoid, we performed immunohistological analyses to evaluate the presence of cell–cell interaction markers, namely, markers for tight junctions (JAM1, Occludin, and Claudin 5), gap junctions (Connexin 26 and 43), and desmosomes (Desmoplakin) as well as for water channel marker aquaporin 4 (AQP4) in retrobulbar, midorbital, and intracanalicular human ON sections.

**Results:**

MECs displayed immunopositivity for markers of tight junctions (JAM1, Occludin, and Claudin 5) and gap junctions (Connexin 26 and 43) as well as for AQP4 water channels. However, no immunopositivity was found for Desmoplakin.

**Conclusion:**

MECs are connected *via* tight junctions and gap junctions, and they possess AQP4 water channels. The presence of these proteins emphasizes the important function of MECs within the ON microenvironment as part of the meningeal barrier. Beyond this barrier function, the expression of these proteins by MECs supports a broader role of these cells in signal transduction and CSF clearance pathways within the ON microenvironment.

## Introduction

The optic nerve (ON) connects the eye to the brain. A variety of ON degenerations such as glaucoma, papilledema, and optic neuritis are associated with damage of the ON resulting in vision loss. A novel concept supports the idea of a possible influence of the ON microenvironment and therefore the meninges as part of this environment in the pathogenesis of these diseases. The meninges consist of three different layers, the dura mater, the arachnoid, and the pia mater. These layers form the subdural space between dura and arachnoid, as well as the subarachnoid space (SAS) between arachnoid and pia mater. The cerebrospinal fluid (CSF) flows intracranially *via* the optic canal into the SAS of the ON that ends as a cul de sac at the lamina cribrosa (1). There is strong evidence that the cellular element of the meninges, the meningothelial cells (MECs) which cover the arachnoid, the pia, and the inner wall of the dura mater as well as the trabeculae and septae within the SAS in the brain and the ON contribute to the homeostasis of the ON microenvironment (2–4). Yet, until now, MECs have only been poorly characterized and not much is known about their function within the ON microenvironment.

It is generally accepted that these cells provide a barrier function between CSF and the brain as well as the neuronal tissue. For this barrier to be efficient, MECs have to form a tight interconnected cellular network. Consequently, a structural hallmark of the MEC layer is cell–cell interaction markers including tight junctions, gap junctions but also desmosomes (5, 6). While tight junctions form a tight barrier and selectively prevent the free passage of solutes and molecules between cells, gap junctions allow for the passage of water, small molecules (<1 kDa), and ions between adjacent cells, thus playing an important role in the maintenance of cellular metabolic activity but also in signal transduction. Desmosomes, on the other hand, are known to be important with regards to contact inhibition as well as cell–cell adhesion (7).

Recent research on MECs demonstrated that these cells not only possess passive barrier function but also seem to be actively involved in a variety of physiological and pathophysiological processes within the SAS. MECs have been shown to react to stimuli such as pressure and oxidative stress with the secretion of cytokines and proteins thus actively contributing to CSF composition (8). Furthermore, MECs have been shown to be highly active phagocytes that are able to ingesting large amounts of bacteria, implicating them in inflammatory processes and antimicrobial host defense (4, 9). With regards to their involvement in eye disease, Pache and Meyer also showed that an enhanced proliferation of MECs and the formation of cell nests are found in ON sections of glaucoma patients compared to healthy controls (10).

Based on our previous work on MECs, the discovery of an ON glymphatic network has opened up the question if MECs might also be involved in CSF/interstitial fluid (ISF) exchange. To this end, we evaluated MECs in retrobulbar, midorbital, and intracanalicular human ON sections of seven patients for the presence of cell–cell interaction markers including tight junctions, gap junctions, and desmosomes as well as aquaporin 4 (AQP4) water channels.

## Materials and Methods

### Human Tissue Samples

The orbital and canalicular portions of both ONs were obtained postmortem after removal of the orbital roof and opening the optic channels. The ONs were removed within (11.0 ± 4.7 h) after death from seven donors (14 eyes; M:F = 7:0; mean age 62.3 ± 12.3 years) without known ophthalmological or neurological disease. The ONs were immediately fixed in paraformaldehyde (4%) and processed for histology. The cause of death in the seven donors was heart failure (three donors), dissecting thoracic aortic aneurysm (one donor), dissecting infrarenal aortic aneurysm (one donor), metastatic small cell lung cancer (one donor), and oropharyngeal cancer (one donor). The ONs were measured in length and diameter and were sectioned into three segments: retrobulbar portion (4 mm retrobulbar), midorbital portion (17 mm retrobulbar), and intracanalicular portion (27 mm retrobulbar). Coronal as well as sagittal sections were prepared and histologically processed. Semiquantitative assessment of marker expression was performed by two blinded observers, the level of immunopositivity was graded between 0 (no expression), 1 (low), 2 (intermediate), and 3 (high). This study was designed and performed in accordance with the Declaration of Helsinki. Written informed consent was obtained as part of the agreement for autopsy.

### Immunohistochemistry

Optic nerve samples including the bulbar, midorbital, and intracanalicular segments were formalin fixed and paraffin embedded. For each segment of human ON samples, 4 μm coronal and sagittal sections were prepared using a microtome. Slides with ON sections were stained with the following antibodies: junctional adhesion molecule A (Novus Biologicals, H00050848-M01), Occludin (abcam, ab31721), Claudin 5 (abcam, ab15106), Connexin 43 (Sigma, C6219), Connexin 26 (abcam, ab38584), and Desmoplakin I + II (Progen, 65146). Antigen retrieval using CC1 buffer (Tris/Borate/EDTA buffer, pH 8.0–8.5) was performed for all antibodies. The extent of cell–cell interaction markers was semiquantitatively determined by eye (scale ranging from 1 to 3). To evaluate specificity of immunohistochemistry staining procedure, control staining (absence of primary antibody) for each marker was performed on coronal sections of human meninges (retrobulbar portion of the ON) (Figure S1 in Supplementary Material).

## Results

**Figure 1:**
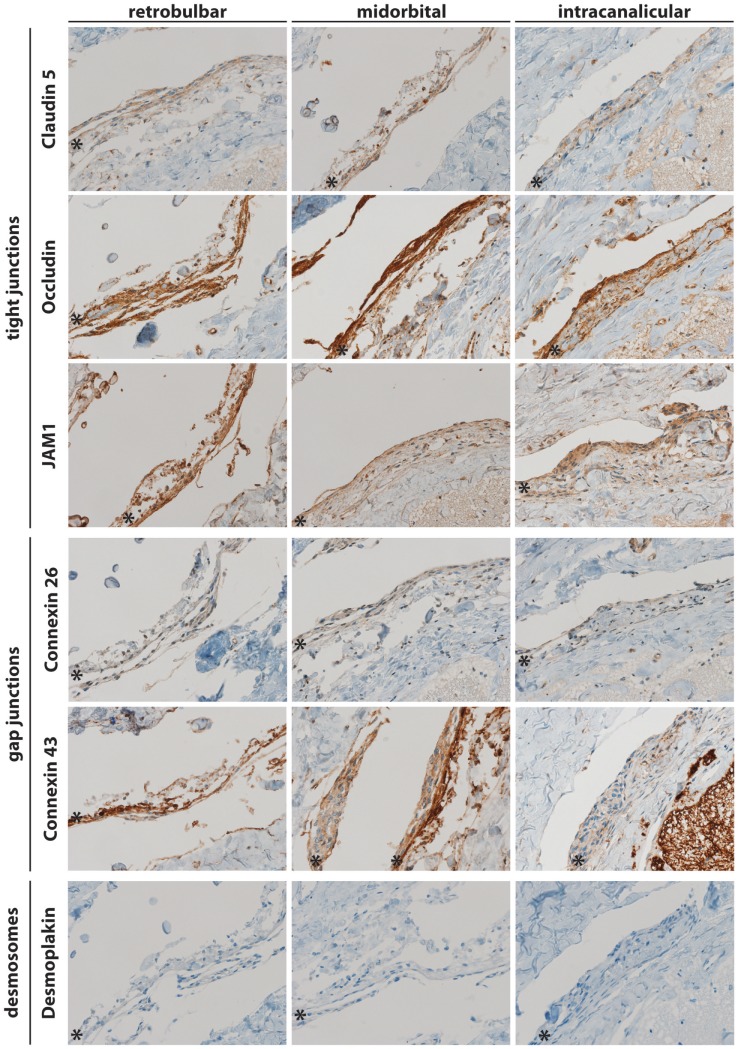
Coronal sections of human meninges including the retrobulbar, midorbital, and intracanalicular part of the optic nerve stained for cell–cell interaction markers of tight junctions (JAM1, Occludin, and Claudin 5), gap junctions (Connexin 26 and 43), and desmosomes (Desmoplakin). A representative staining of one patient is shown. * indicates arachnoid layer.

**Figure 2:**
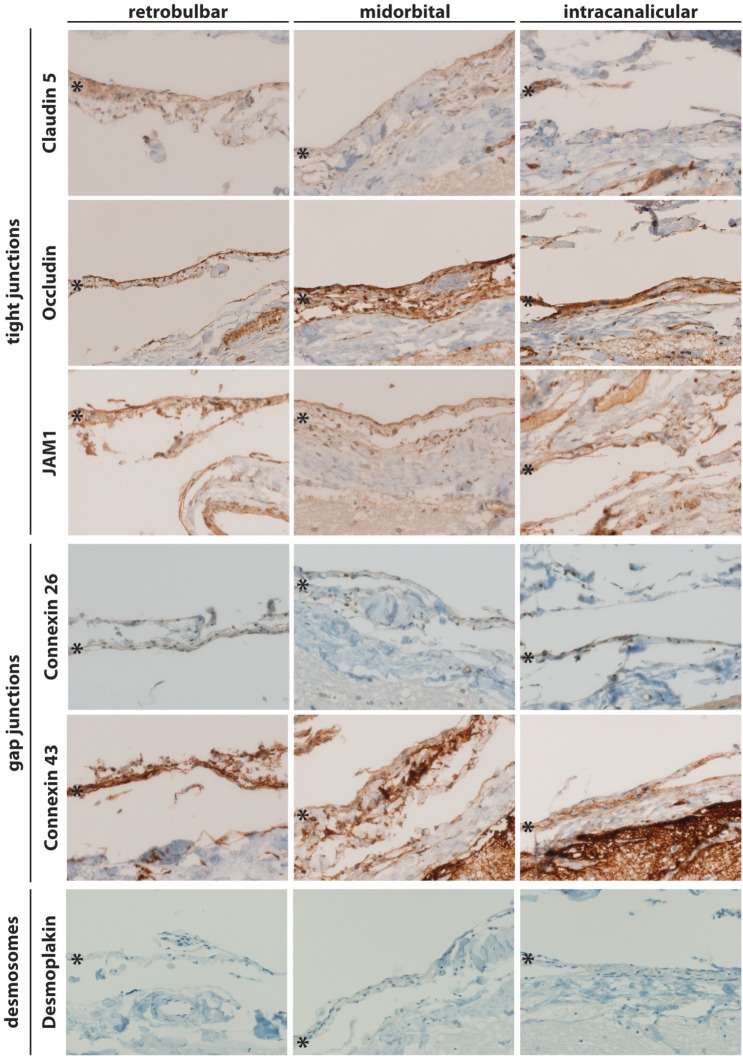
Sagittal sections of human meninges including the retrobulbar, midorbital, and intracanalicular part of the optic nerve stained for cell–cell interaction markers of tight junctions (JAM1, Occludin, and Claudin 5), gap junctions (Connexin 26 and 43), and desmosomes (Desmoplakin). A representative staining of one patient is shown. * indicates arachnoid layer.

For a better understanding of the anatomy of meninges and their cellular component, MECs within the ON, cell–cell contacts were analyzed by immunohistochemistry. To this end, coronal and sagittal sections at retrobulbar, midorbital, and intracanalicular locations of the ON from 14 human eyes (left and right eyes of seven donors) were obtained and stained for the tight junction markers such as Claudin 5, Occludin, and JAM1, the gap junction markers such as Connexin 26 and Connexin 43 as well as the desmosomal marker such as Desmoplakin. As shown in Figure 1, Claudin 5 was found in the arachnoidea and to some extent in the subarachnoidal area. Similarly, Occludin (Figure 1, panel 2) and JAM1 (Figure 1, panel 3) immunopositivity was found in the arachnoidal and subarachnoidal locations. These data are consistent with the expression of tight junctions between MECs. As for gap junctions, Connexin 26 and Connexin 43 immunoreactivity was found in the arachnoid and in subarachnoidal locations along the entire ON (Figure 1, panels 4 and 5). Interestingly, the desmosomal marker Desmoplakin was not detected in any location along the ON (Figure 1, panel 6). To confirm these findings, sagittal sections from locations along the ON were also analyzed by immunohistochemistry (Figure 2). As in the coronal sections, the tight junction markers, such as Claudin 5, Occludin, and JAM1, were found in the arachnoid and in subarachnoidal locations along the ON. Similarly, immunopositivity for the gap junction markers, such as Connexin 26 and 43, was found retrobulbar, midorbital, and intracanalicular.

**Figure 3:**
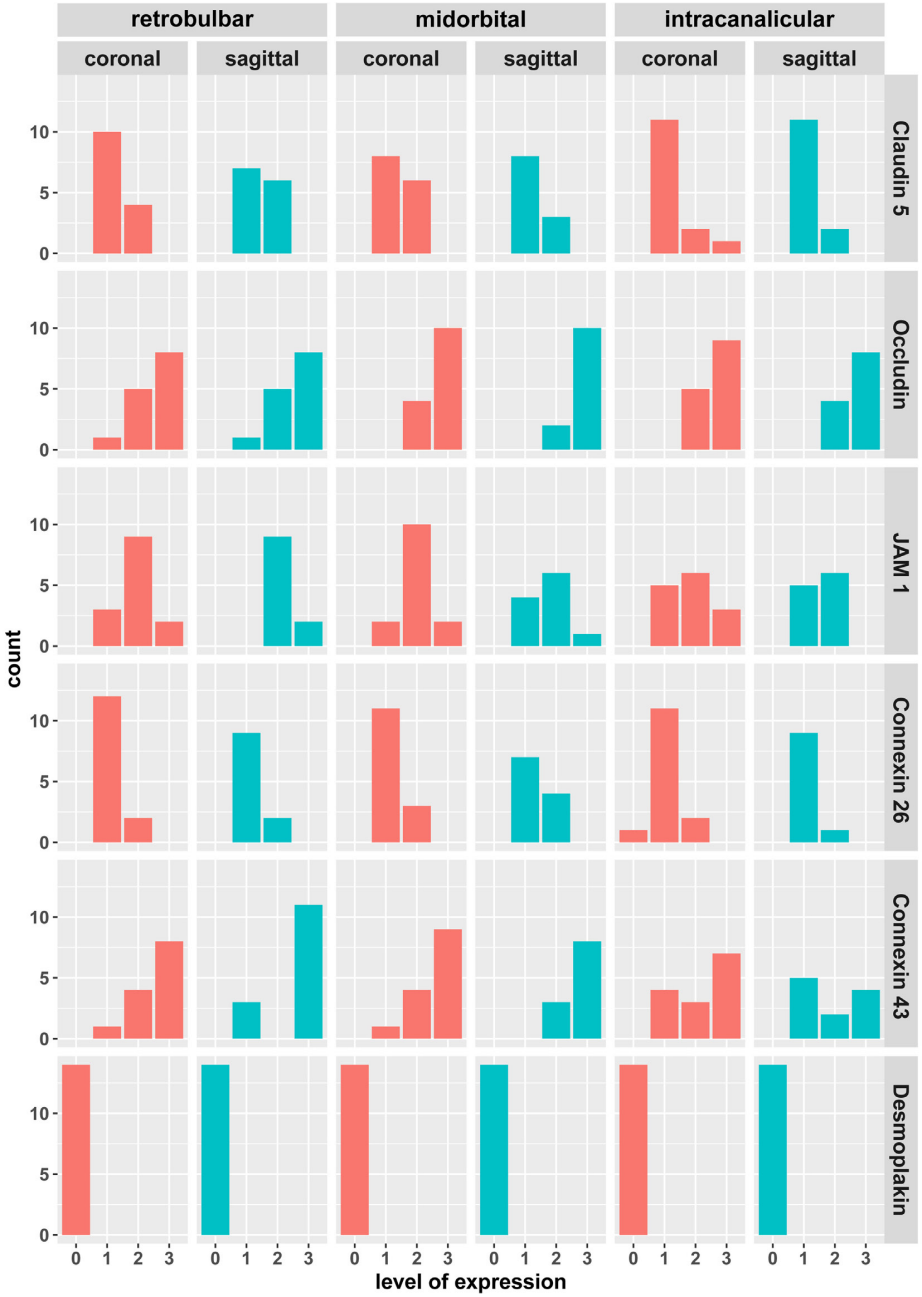
Semiquantitative assessment of the coronal and sagittal sections of human meninges including the retrobulbar, midorbital, and intracanalicular part of the optic nerve stained for cell–cell interaction markers of tight junctions (JAM1, Occludin, and Claudin 5), gap junctions (Connexin 26 and 43), and desmosomes (Desmoplakin). Scale for expression evaluation ranging from 0 (no immunopositivity) to 3 (high immunopositivity).

To assess intra-patient variability of cell–cell contact expression, the level of immunopositivity of each marker protein was compared between samples from 14 ONs from 7 different patients again at retrobulbar, midorbital, and intracanalicular locations. Following independent assessment by two blinded observers, the level of immunopositivity was graded between 0 (no expression), 1 (low), 2 (intermediate), and 3 (high). As shown in Figure 3, Claudin 5 expression is low to intermediate in all samples analyzed. Similarly, Connexin 26 is consistently low between the samples. As for Occludin, JAM1, and Connexin 43, expression differs between samples with Occludin showing a trend toward mainly high expression. As for Desmoplakin, no immunoreactivity was detected in all 14 samples analyzed.

**Figure 4:**
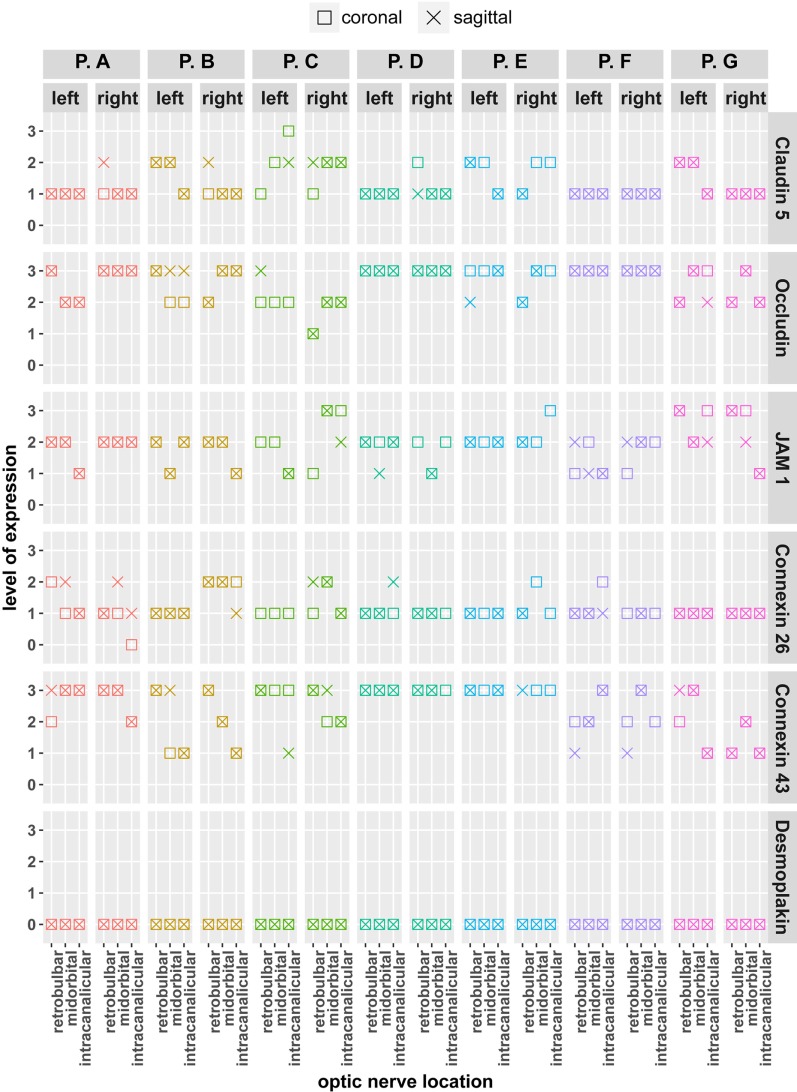
Comparison of expression levels of cell–cell interaction markers of tight junctions (JAM1, Occludin, and Claudin 5), gap junctions (Connexin 26 and 43), and desmosomes (Desmoplakin) with regard to patient sample for coronal and sagittal sections. For each patient, left and right eyes were compared. Scale for expression evaluation ranging from 0 (no immunopositivity) to 3 (high immunopositivity). Marker levels between different samples (left/right eye, coronal/sagittal, and retrobulbar/midorbital/intracanalicular) of a given patient were not apparently different.

To further assess our findings, immunopositivity of markers of cell–cell contact was evaluated on the patient, eye, location, and sample level. Figure 4 summarizes our findings for each left and right eyes of each patient. While there are differences in the level of certain markers between individual patients, marker levels between different samples (left/right eye, coronal/sagittal, and retrobulbar/midorbital/intracanalicular) of a given patient were not apparently different.

**Figure 5:**
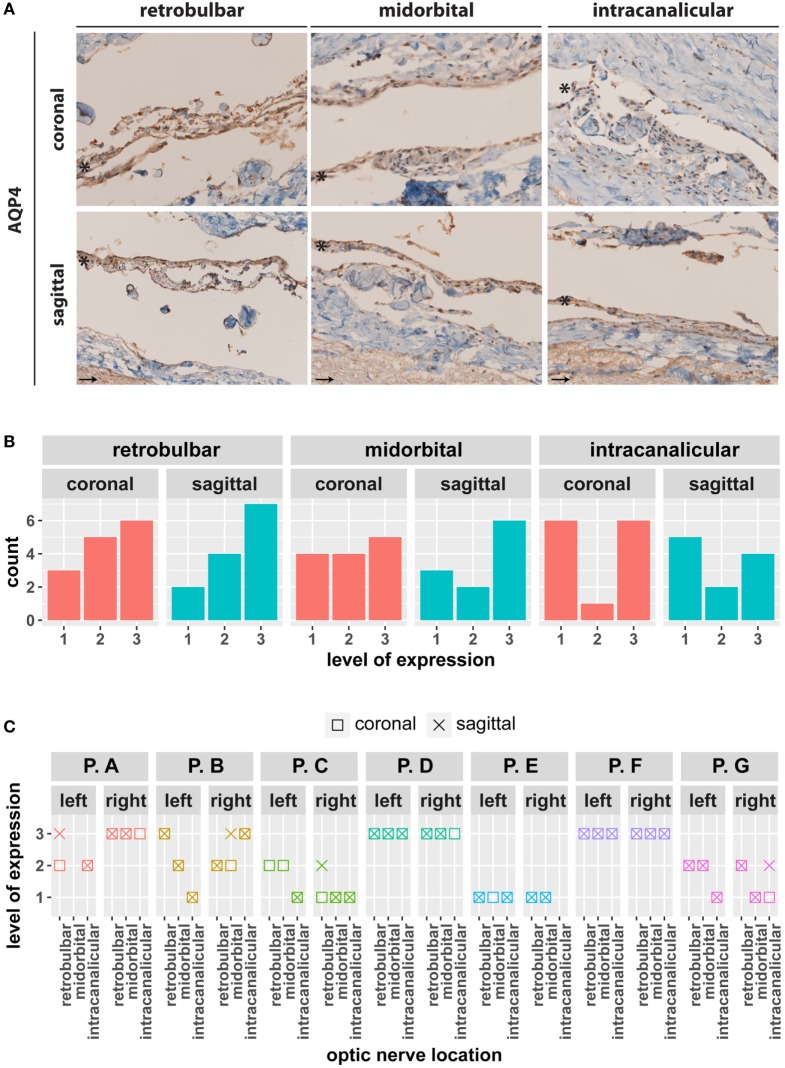
**(A)** Coronal and sagittal sections of human meninges including the retrobulbar, midorbital, and intracanalicular part of the optic nerve (ON) stained for water channel marker aquaporin 4 (AQP4). A representative staining of one patient is depicted. * indicates arachnoid layer; → indicates ON tissue.**(B)** Semiquantitative assessment of the coronal and sagittal sections of human meninges stained for AQP4. **(C)** Comparison of expression levels of AQP4. Scale for expression evaluation ranging from 0 (no immunopositivity) to 3 (high immunopositivity). Marker levels between different samples of a given patient were not apparently different.

So far, identification of cell–cell contacts between MECs in the ON underlines the role of meninges in barrier formation. However, biological barriers serve not only to separate but also to allow selective exchange between two compartments. Therefore, the expression of AQP4 in meninges of the ON was analyzed by immunohistochemistry. As shown in Figure 5, AQP4 immunoreactivity was found in the arachnoid and subarachnoidal locations in retrobulbar, midorbital, and intracanalicular locations. Aquaporin expression seemed to vary between different patients (Figure 5B); however, individual analysis (Figure 5C) revealed similar expression levels between samples from the same patient. AQP4 immunoreactivity was found as well in the ON tissue where it is known to be expressed in astrocytes.

## Discussion

The meninges cover and protect the brain, the spinal cord, and the ON form mechanical injury and provide a space for CSF. They also play a role in supplying blood to the brain. Our evaluation of MECs within human ON sections applying immunohistochemistry demonstrated expression of tight junction markers, gap junction markers, and AQP4 water channels. Regarding tight junction markers, we found expression of Claudin 5, occluding and JAM1 within the meninges of the ON sections in all patients. The presence of tight junctions within the meninges of the brain is well known and has been previously described in electron microscopy studies. These studies revealed that in brain meninges such junctions are found especially in cells at the border of the arachnoid with the dura affirming their function of restricting diffusion of solutes across intercellular spaces and thereby posing a physical barrier to CSF (5). Concerning gap junction markers in the ON meninges, we found immunopositivity for Connexin 26 and 43. Gap junctions are specialized intercellular junctions that facilitate cell–cell communication by acting as a selective transporter of small molecules and ions between cells. It has been previously shown that cells expressing gap junctions can act as a bystander to neighboring cells by distributing toxic load *via* these junctions, thus limiting the impact of toxic substances on the single cell level (11). It is therefore conceivable that MECs might act in a similar fashion by taking up waste substances from the CSF shuttling them along the MEC layer toward the glymphatic network. Although the presence of desmosomes has been described within the MEC layer in the brain (6), we did not find Desmoplakin immunopositivity within the arachnoid layer in the ON.

The glymphatic system is a brain-wide paravascular route that mediates CSF/ISF exchange and thereby supports the clearance of solutes from the brain (12). The ON is a white matter tract of the brain extending into the orbit *via* the optic canal; therefore, the presence of such a system in the ON has been suspected. There has been increasing evidence of the existence of lymphatic structures in murine and also human dura mater of the brain and the ON (1, 13–17). The first histological evidence for a paravascular pathway within the ON has been shown in a recent publication. However, how exactly the CSF/ISF exchange, and especially the drainage of CSF from the SAS into the lymphatic network, is realized is still not completely understood. As MECs cover the entire SAS and form a cellular barrier between the SAS and the interstitial space, MECs could be involved in waste shuttling and drainage of CSF into the lymphatic network by means of cell–cell junctions and AQP4 water channels (18).

In addition to cell–cell interaction markers, we therefore investigated the ON meninges for the presence of water channel marker AQP4. Aquaporins are membrane proteins that have been mainly implicated in epithelial fluid transport (19). Iliff et al. recently showed that AQP4-expressing astrocytes that surround the cerebral capillaries contribute to CSF flux into the parenchyma thereby facilitating the clearance of interstitial solutes from the brain interstitium (20). As described for astrocytes, one could speculate that AQP4-expressing MECs might as well promote solute transport across the MEC layer thereby contributing to CSF flux across the meninges into the glymphatic system.

In summary, the presence of tight junctions, gap junctions, and AQP4 on MECs provides new evidence that MECs might also play a role in CSF clearance pathways within the ON microenvironment and the brain.

## Ethics Statement

This study was carried out in accordance with the recommendation of “Guidelines of the Department of Pathology, Kantonsspital Aarau and the Ethics Committee Nordwestschweiz” with written informed consent from all subjects. All subjects gave written informed consent in accordance with the Declaration of Helsinki. The protocol was approved by the “Ethics Committee Nordwestschweiz.”

## Author Contributions

TZ together with CK performed the main part of the practical laboratory work and analyses of the results. AN made intellectual contributions and participated in analyses of the results. HK and PM made intellectual contributions and supervised the work. TZ, CK, and PM wrote the manuscript.

## Conflict of Interest Statement

The authors declare that the research was conducted in the absence of any commercial or financial relationships that could be construed as a potential conflict of interest.
