# Supplementary material for: Cell–Cell Interaction Proteins (Gap Junctions, Tight Junctions, and Desmosomes) and Water Transporter Aquaporin 4 in Meningothelial Cells of the Human Optic Nerve
Source: Front Neurol. 2017 Jun 29;8:308. doi: 10.3389/fneur.2017.00308 (PMC5489558; doi:10.3389/fneur.2017.00308)
Supplement: Supplementary file 1 [file Image_1.PDF]

*Supplementary Material*

# Cell-Cell Interaction Proteins (Gap-Junctions, Tight-Junctions, and Desmosomes) and Water Transporter Aquaporin 4 in Meningothelial Cells of the Human Optic Nerve

**Thi Ngoc Co Zeleny[#], Corina Kohler[#], Albert Neutzner, Hanspeter E. Killer[*], Peter Meyer[*]**

# Authors contributed equally to this work

*** Correspondence:**

Corresponding Authors: peter.meyer@usb.ch and hanspeter.killer@ksa.ch

**Supplementary Figures**

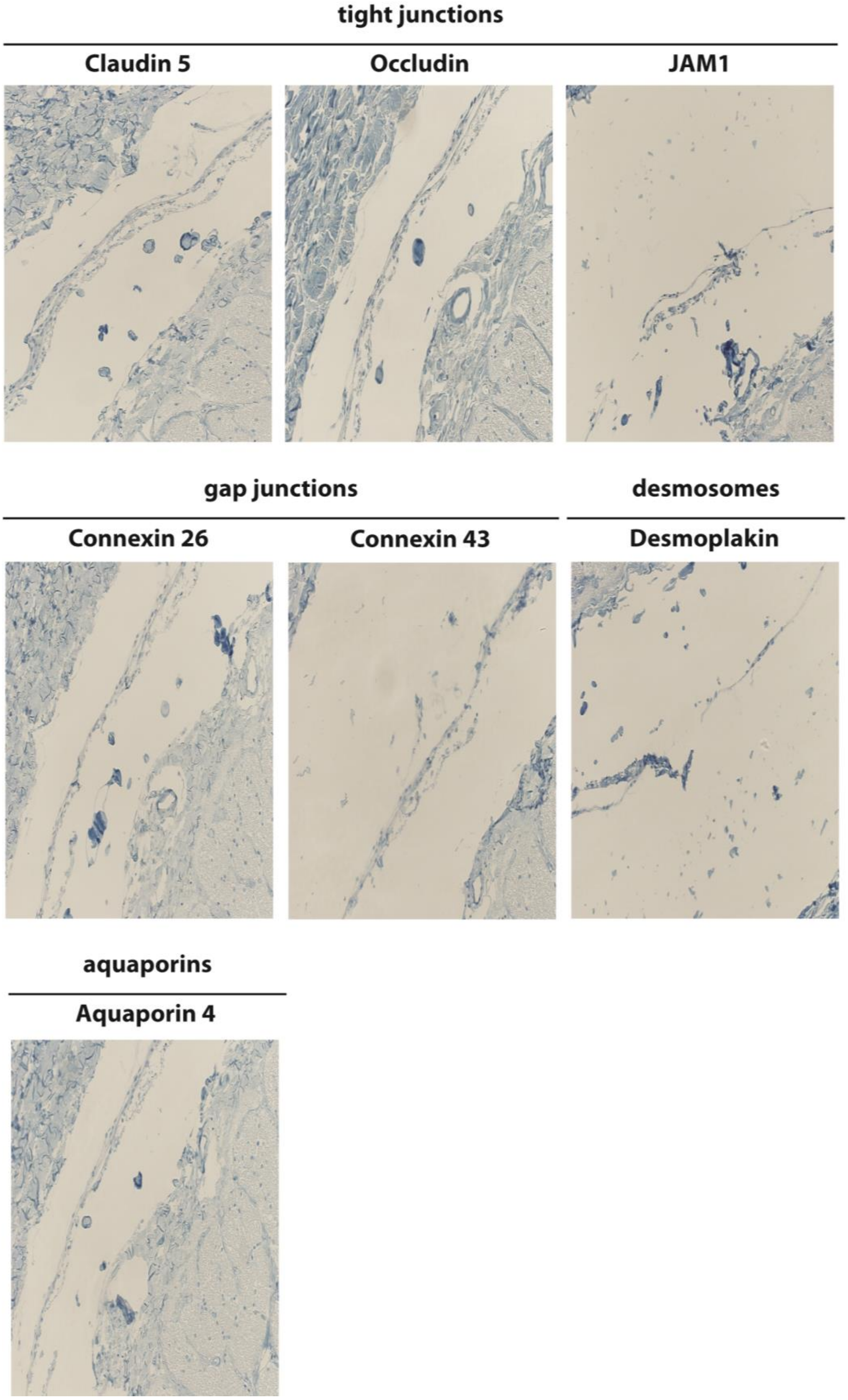


**Supplementary Figure 1:** Immunohistochemistry staining control. To evaluate specificity of IHC staining procedure, control staining (absence of primary antibody) for each marker was performed on coronal sections of human meninges (retrobulbar portion of the optic nerve).
